# Electrospun Sound-Absorbing Nanofibrous Webs from Recycled Poly(vinyl butyral)

**DOI:** 10.3390/polym14225049

**Published:** 2022-11-21

**Authors:** Petr Filip, Tomas Sedlacek, Petra Peer, Martin Juricka

**Affiliations:** 1 Institute of Hydrodynamics, Czech Academy of Sciences, 166 12 Prague, Czech Republic; 2 Centre of Polymer Systems, Tomas Bata University in Zlin, 760 01 Zlin, Czech Republic; 3 Department of Polymer Engineering, Faculty of Technology, Tomas Bata University in Zlin, 760 01 Zlin, Czech Republic; 4 Department of Physics and Material Engineering, Faculty of Technology, Tomas Bata University in Zlin, 760 01 Zlin, Czech Republic

**Keywords:** poly(vinyl butyral), poly(vinyl butyral) recycling, electrospinning, sound-absorption

## Abstract

The amount of poly(vinyl butyral) (PVB) foils added to car windscreens to suppress glass shattering represents a huge worldwide volume of the material, and once a vehicle is end-of-life it also becomes a significance contributor to landfill. The recycling of PVB materials from windscreens has been expensive and despite improvements in recycling technologies, the landfill burden still increases. However, an increase in oil prices can shift the economic balance and stimulates the possible applicability of recycled PVB. As PVB is a relatively easy electrospinnable material, it is shown that nanofibrous mats produced from recycled PVB blends in ethanol exhibit very good sound-absorbing properties. To achieve an optimal composition between virgin and recycled PVB blends, a series of their ratios was consecutively characterized using various techniques (rheometry, SEM, FTIR, DSC, TGA, DMA, an impedance tube for determining sound absorbance). The best result was obtained with two wt. portions of 8 wt.% solution of virgin PVB in ethanol and one wt. portion of 12 wt.% solution of recycled PVB in ethanol.

## 1. Introduction

**Figure 1:**
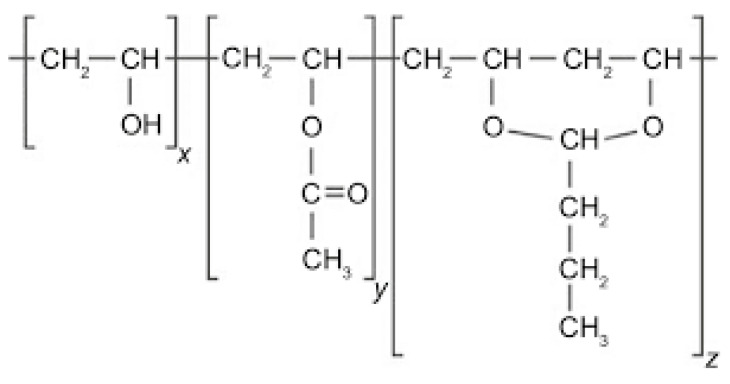
Chemical structure of the terpolymer poly(vinyl butyral) containing butyral (z ≈ 0.8) and hydroxyl (x ≈ 0.2) side groups with a small amount of acetate (y ≈ 0.01) units.

From the very beginning, the introduction of terpolymer poly(vinyl butyral) (PVB, Figure 1) to industrial production (invented in 1927 [1]) has had an application dominant within the automotive sector. More specifically, PVB sheets are used as the interlayer material in glass windscreens, based on their properties such as optical clarity, toughness, and flexibility. A crucial factor is also good adhesion to various substrates including glass (after the laminating process). On the other hand, weather conditions exhibit an adverse contribution to the quality of PVB interlayers. Their adhesion to glass is impaired by ambient humidity, and ultraviolet radiation contributes to a decrease in mechanical properties [2]. At present, the usage of PVB in automotive applications forms approximately two thirds of its worldwide production. In spite of the fact that laminated glass constitutes up to 3% of the total material in end-of-life vehicles (ELV) every year, it still represents about 500,000 tons [3].

This measure, together with higher land costs and the rise in the price of raw materials, makes recycling windscreens attractive since the standard practice of burying them in landfill (with increasing fees) is no longer sustainable [4,5,6]. The recycling of laminated glass windscreens provide glass (the easiest component for recycling), silica [7], and PVB.

There are two aspects in recycling PVB:

(1) The production of new PVB material is relatively cheaper than obtaining recycled PVB from the windscreen foils. However, in this case, it is necessary to take into account other factors such as increasing landfill fees, increasing oil prices, and the impact on the environment. After subtracting these items from the gross processing costs, the reprocessing of re-cycled PVB becomes still more attractive, with acceptable net charges [8,9].
(2) Apart from PVB glass scrap (caused by hydroxyl groups along the polymer and silanol groups on the glass surface [10]), and water impeding the interlayer recycling, the recycling process also contains plasticizers used during PVB sheet production. This problem is intensified by the fact that different manufacturers use different types and quantities of plasticizers [11]. Among plasticizers, dibutyl sebacate is dominantly used (20–25% according to Dhaliwal and Hay [9] and also confirmed by Sonego et al. [12]).

Therefore, it is necessary to find applications for waste poly(vinyl butyral) (w-PVB) material, where recycling costs will be at least balanced. In other words, a suitable high value-added materialization strategy should be developed [8]. In this context, up to now, there is only one global manufacturer producing interlayer sheets with a 100% recycled PVB (Butacite G made by Trosifol from Kuraray Group) (Martin et al. [13]).

It is well-known that w-PVB derived from ELV windscreens is one of the most difficult waste resources to recycle [14]. So far, various methods have been proposed. A facile combined approach of selective dissolution and an evaporation method was developed by Wang et al. [15]. Swain et al. [15] applied mechanical separation through hydrodynamics, ultra-sonication assisted separation, and a mechano-chemical separation process. A three-stage technology was proposed by Tupy et al. [11] for recycling PVB sheets. In the first stage, laminated safety glass was mechanically cracked. In the second stage, the adhesion reduction to a minimal degree was followed by a chemical-physical assisted separation. This causes a self-release from the glass out of the PVB sheet. The third stage was the mechanical peeling of the remaining glass from the PVB sheet, which completed the recycling process. This was introduced after the reprocessing of the PVB; the obtained interlayer showed a decrease in its light transmission value; the light transmission drops by about 0.2% after every reprocessing cycle. To extract plasticizers from recycled PVB, Wang et al. [16] applied supercritical fluids.

Recycled PVB (rPVB) found its place in various applications due to its properties, see Table 1.

All these applications show that the unique properties of rPVB have excellent utility. However, there is still a choice of electrospinning methods, including the least expensive, which would apparently reduce the acquisition costs of the resulting product. The electrospinning process is based on emitting viscoelastic polymer jets under a high-voltage electric field (in orders of tens kV), which polymer solutions are exposed to. During the passage (≈15–30 cm) of the jets to a grounded collector, solvent evaporates and ideally pure polymeric material impacts in the form of nanofibres forming non-woven webs. This process was used by Park et al. [29] for producing carbon nanofibres from composites of rPVB and natural cellulose. Nitrogen-doped carbon nanofibers, with open channels using polyacrylonitrile, rPVB and urea, were prepared by Park et al. [8].

A possible use of rPVB can be also directed to sound absorbing materials, such as traffic silencing, serving to suppress the noise of electric motors and anti-noise barriers or inside earmuffs [30]. Their advantage in controlling noise intensity is long-term and for the lifetime of the product, benefiting from negligible maintenance costs and consistent performance. Electrospun porous sound absorbing materials can be easily formed and are light weight. These materials are used as a decorative complement to classical acoustic materials and can efficiently contribute to the broadening of absorption frequency range.

**Figure 2:**
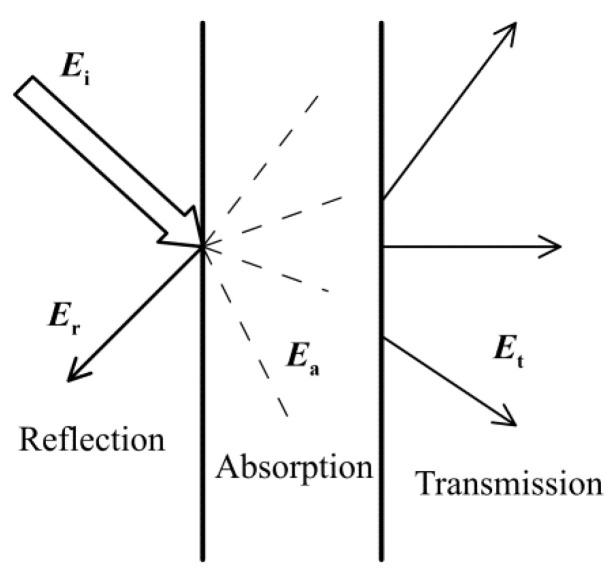
Distribution of the incident energy.

After surface impact on the material, the sound energy is separated into three components:

*U*_i_ = *U*_r_ + *U*_a_ + *U*_t_, (1)

where *U*_i_ is the total incident energy and *U*_r_, *U*_a_, and *U*_t_ successively represent the reflected, absorbed, and transmitted components, see Figure 2. For sound absorbing materials, the dominant role of the absorbed energy component is expected, and the so-called sound absorption coefficient (SAC) is defined as a ratio *U*_a_/*U*_i_.

The magnitude of the SAC parameter is the result of three main principles [31,32]:

- vibrating air molecules in nanofibrous materials rubbing with the individual nanofibres, which results in the conversion of sound energy into heat and consequent dissipation;
- the penetration of longitudinal sound waves, accompanied by periodic compression and the release of air in the pores contributing to energy transformation; this mechanism is further enhanced by the interconnection of the nanopores enabling the diffusion of sound waves into the whole nanofibrous mats;
- the resonance of individual nanofibres converting sound energy into mechanical and heat energy.

This process is graphically depicted in Rahimabady et al. [31], where the energy of sound waves is processed through viscous and thermal effects, and material damping.

In this respect, the attributes specific for nanofibrous materials favors the above mentioned conversion of incident sound energy. The characteristic high surface-to-volume ratio has a positive impact on rubbing areas; a small nanofibre diameter contributes to better resonance, and hence to sound energy attenuation [33].

The complex sound absorption mechanism in nanofibrous materials is affected, among others, by:

- thickness (prolonging the dissipation path of sound waves and enlarging their contact area with the nanofibres);
- the degree of material elasticity contributing to better resonance;
- the value of the nanofibre diameter strongly influencing a surface-to-volume ratio and the porosity of the mats;
- the quality of the surface of nanofibers; their beadless character improves efficiency of sound energy absorption.

Based on the preceding analysis, the aim of this contribution is to optimize the rPVB nanofibers prepared by electrospinning across blends of virgin and recycled rPVB-ethanol solutions. The quality of the individual nanofibers will be evaluated according to a smoothness of their surface (suppressing the appearance of unwanted beads abruptly changing nanofibers diameters) and sound absorption will be optimized with respect to the composition of blends.

## 2. Materials and Methods

### 2.1. Materials

The virgin PVB (Mowital B 45H, the suffix H indicates the degree of acetalization, *M*_w_ = 45,000 g.mol^−1^, Kuraray Europe Moravia s.r.o., Holesov, Czech Republic) is a random terpolymer containing butyral and hydroxyl side groups with a small amount of acetate units [9]. In this case vinyl butyral, vinyl alcohol and vinyl acetate participate by 75–81, 18–21 and 1–4%, respectively. Virgin PVB and ethanol (quality of p.a., Penta, Prague, Czech Republic) were used as obtained without further refinement. The recycled poly(vinyl butyral) (rPVB) based on unspecified extrusion types supplied by the recycling company Sklopan Liberec, a.s. (Liberec, Czech Republic) was collected by the dry method from the automotive windscreens waste [11].

### 2.2. Solutions Preparation Using Virgin, Recycled and Blends of PVB

Each polymer (virgin vPVB and recycled rPVB) at concentrations of 8, 10, and 12 wt.% was dissolved separately in ethanol using a magnetic stirrer (Heidolph MR Hei-Tec, Schwabach, Germany) under constant conditions (mixing rate 250 rpm and temperature 25 °C for 48 h). Consequently, bPVB (PVB blends) consisted of rPVB and vPVB solutions (weight ratios 2:1, 1:1, and 1:2) were magnetically stirred for 2 h at the temperature of 25 °C. The chosen notation is introduced in Table 2.

### 2.3. Characterisation of PVB Solutions (Rheology, Electric Conductivity)

The quality of the resultant nanofibrous webs is influenced by three groups of entry parameters: material (characteristics of source material, solvent, and solution), processing (voltage, conductivity, collector distance, etc.) and environmental (temperature, humidity). An emphasis was given to two factors:

(a) conductivity participates in the stretching of viscoelastic jets as a result of higher levels of charge carried by the solution. This participates in the possible appearance of beads along the nanofibers—singularities characterized by the abrupt increase in the nanofibers diameter followed by its sudden reduction. The electric conductivity of the polymer solutions was defined with a Conductivity Meter Lab 960 device (SCHOTT Instruments, Mainz, Germany);
(b) rheological characterization indicating after electrospinning the possible appearance of ‘blobs’ (merged wet agglomerates—abundance of solvent), regular nanofibrous (solvent evaporated), or passage to electrospraying (disintegration into individual drops—insufficient amount of solvent). Rheological characteristics were evaluated using a Physica MCR 501 rotational rheometer (Anton Paar, Graz, Austria), equipped with the concentric cylinders (26.6/28.9 mm inner/outer diameters) at the constant temperature of 25 °C. The linear viscoelastic region (LVE) was determined applying oscillatory measurements (varying frequency and strain), shear viscosity was measured in the range 0.01–300 s^−1^. Applied shear viscosity was measured at the shear rate of 0.12 s^−1^ (within the LVE region). Each measurement was repeated at least three times with very good reproducibility.

### 2.4. Electrospinning of PVB Solutions

The nanofibrous webs were electrospun from prepared PVB solutions using our lab-made needleless apparatus, for details see [34]. The voltage between a semi-spherical pit (0.2 mL) terminating the carbon steel stick (10 mm in diameter) and a grounded collector (aluminum foil) was generated by a high voltage power supply (Spellman SL70PN150, Hauppauge, NY, USA). The setting of the process parameters: voltage 20 kV, a tip-to-collector distance attained 10 cm, ambient temperature 23 ± 1 °C, relative humidity 40 ± 1%.

### 2.5. Characterisation of Nanofibrous Webs (SEM, FTIR, DSC, TGA, DMA, WCA)

Relatively apparent characterization of virgin PVB will contrast to that for recycled PVB which contains various impurities that are potentially connected with the PVB foils processing and exacerbated by the recycling procedure of the windscreens. Hence, for proper characterization of the electrospun nanofibrous webs, a deeper analysis is inevitable. In the following, the individual devices and experimental procedures used for this aim are described.

Prior to applying a high-resolution scanning electron microscope (SEM) Vega 3 (Tescan, Brno, Czech Republic) the nanofibrous webs were sputtered by a conductive gold layer using a sputter Quorum Q150R (Quorum Technologies Ltd., Lewes, UK) to improve conductivity. Adobe Creative Suite software was used for a determination of the mean diameter based on processing of 300 nanofibers from 3 different images and of the mean pore size calculated from 30 values.

The presence of specific chemical groups in the tested nanofibrous webs was analyzed with the help of a Fourier Transform Infrared (FTIR) spectrometer Nicolet iS5 (Thermo Scientific, Waltham, MA, USA) equipped with the ATR accessory accommodated with the germanium crystal. The FTIR experiments were performed in the attenuated total-reflection mode at an ambient laboratory temperature. The spectra were collected in a wavelength region from 4000 to 680 cm^−1^ using 64 scans with a spectral resolution of 2 cm^−1^. The obtained FTIR spectra of tested nanofibrous webs were normalized and major vibration bands were associated with the typical chemical groups.

Glass transition temperature, *T*_g_, of webs was determined using a differential scanning calorimeter (DSC) (DSC 1, Mettler Toledo, Greifensee, Switzerland). For the thermal analysis heating/cooling/heating/cooling temperature ramps with heating/cooling rate of 20 °C/min (to promote the visualization of heat capacity changes) were applied in the range between −10 to 130 °C under protective nitrogen atmosphere preventing material degradation. The tested specimens of weight of approximately 3 μg cut out directly from the produced nonwoven webs were manually mildly pressed into the bottom of DSC pan using a polytetrafluorethylene (PTFE) ram.

Thermal stability of used materials through thermogravimetric analysis (TGA) was evaluated by the help of a thermogravimeter TGA Q500 (TA Instruments, New Castle, DE, USA) in a temperature range of 25–600 °C at a heating rate of 10 °C/min under air atmosphere (justifying possible application in common practice). Derivative thermogravimetric analysis (DTG) was applied for easier interpretation of the obtained results.

Dynamic mechanical analysis (DMA) describing viscoelastic properties of studied specimens was carried out in temperature range 10–110 °C (heating rate of 5 °C.min^−1^) using a dynamic mechanical analyzer DMA861 (Mettler Toledo, Greifensee, Switzerland) equipped with clamps for tensile testing mode. Since electrospun vPVB nanofibrous webs failed in this analysis completely, two types of specimens (area of each cut out specimen 18 mm × 3 mm) were compared and characterized: electrospun nanofibrous webs of approximate thickness 1 μm and manually hot pressed (at temperature of 120 °C) thin films of approximate thickness 10 μm. While nonwoven webs specimens containing rPVB had a tendency to rupture at temperatures exceeding 85 °C during dynamic mechanical testing, an upper temperature limit was set to 80 °C. Based on the preliminary results of amplitude dependent viscoelastic characterization the linear viscoelastic region (deformation amplitude of 10 μm (0.1%) and frequency of 1 Hz) was defined and applied for further experiments in the specified temperature areas:

- rPVB containing web specimens in the range 10–80 °C;
- thin films of virgin PVB in the range 30–110 °C.

Sound absorption properties were measured at room temperature by means of the measuring set consisting of a BK 4206 two-microphone impedance tube, a BK 3560-B-030 three-channel signal pulse multianalyzer and a BK 2706 power amplifier (all from Brüel and Kjær, Virum, Denmark). Frequency dependencies of the normal incidence sound absorption coefficient α = α(*f*) (relating absorbed and incident sound intensities) over the frequency range of 100–6400 Hz were measured by the transfer function method based on the partially standing wave principle.

Wettability of the samples was evaluated through the water contact angle (WCA) measurements using the Surface Energy Evaluation System (SEE System; Advex Instruments, Brno, Czech Republic). The apparatus was used to visualize the drop’s tangent (right and left) and the three-phase points. Each representative contact angle was calculated by the averaging of at least ten separate readings for each sample. As a testing liquid, deionized water was used, and digital images of a 5 µL water droplet on the surface of pressed films of vPVB and rPVB were captured by the charge-coupled device camera system.

## 3. Results and Discussion

**Figure 3:**
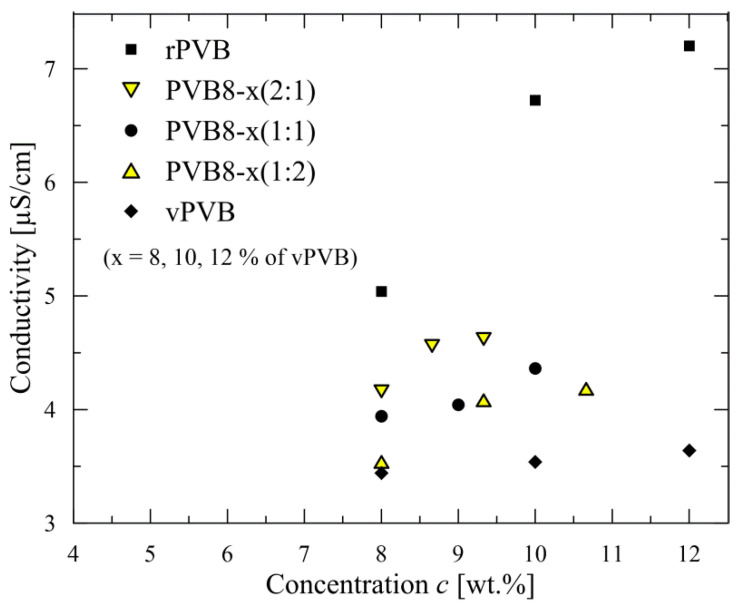
Conductivity of recycled, blended, and virgin PVB.

As already mentioned in the Introduction, recycled PVB contains plasticizers and residues of glass scrap. This reflects in significantly different properties of rPVB in comparison with vPVB, as documented in Figure 3, in the course of conductivity. The presence of impurities in rPVB nearly doubles the value of conductivity in comparison with vPVB (see details in Figure 3). Consequently, the conductivity of blended PVB copies this trend, i.e., conductivity increases with an increasing content of rPVB.

**Figure 4:**
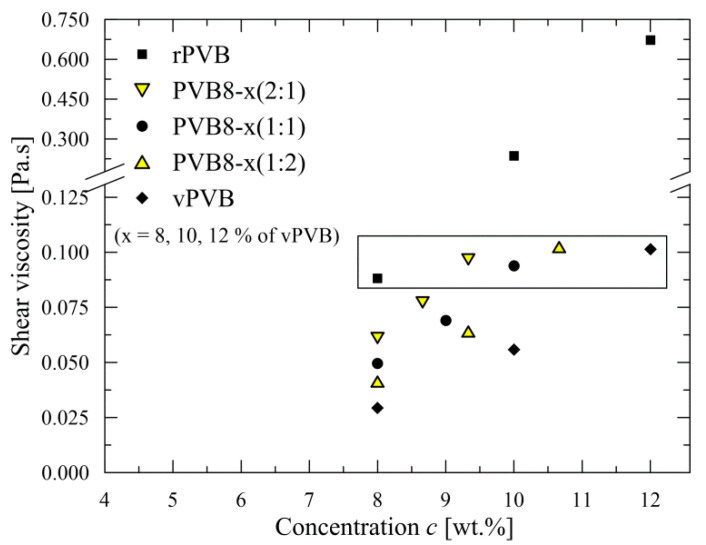
Dependence of shear viscosity on participation of impurities in rPVB.

The quality of nanofibers is strongly subject to the viscosity of electrospun polymer solution [35,36], not only in the appearance of singularities (so called beads), but also in the overall morphology including a mean nanofiber diameter (for the case of vPVB see Yener and Yalcinkaya [37]). For higher values of viscosity, a mean diameter can exceed 1 μm; however, in this case, a fibrous mat loses the advantages of nanofibrous mat, first of all an extremely high ratio relating surface area to occupied volume. A difference in shear viscosities for rPVB and vPVB is depicted in Figure 4 (with the broken ordinate). A considerable difference between rPVB and vPVB reflects -as expected- in a mean nanofiber diameter, as will be presented later. As the value of viscosity (≈0.1 Pa.s) for rPVB solution (8 wt.%) roughly corresponds to that for vPVB solution (12 wt.%), the viscosities for the blends PVB8-12 attain the values between these two viscosities for all weight ratios (2:1, 1:1, 1:2); all five values are depicted in the rectangle in Figure 4. This finding indicates that recycled PVB polymers exhibit a higher average molecular weight and/or the presence of impurities, leading to an increase in viscosity. Notably similar viscosity levels also participate similarly in nanofiber cross sections, as discussed below.

**Figure 5:**
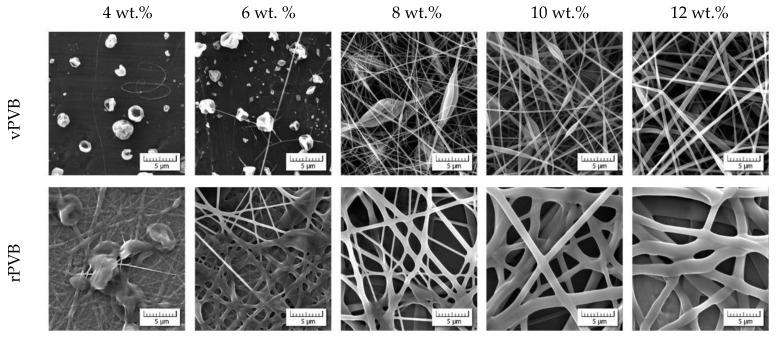
Morphology of nanofibrous webs of recycled and virgin PVB (various concentrations in ethanol).

**Figure 6:**
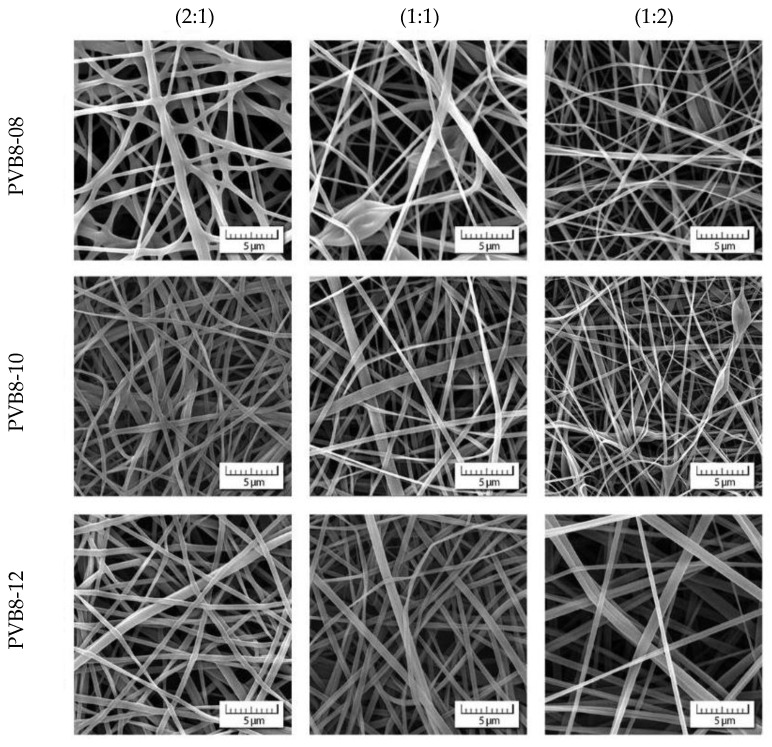
Morphology of nanofibrous webs of blended PVB.

Scanning electron microscopy determined a choice of weighted concentrations of recycled and virgin PVB, see Figure 5. Lower concentrations (4 and 6 wt.%) exhibit rather poor quality of nanofibers, if any. The concentration 8 wt.% represents the starting point for which the nanofibrous webs can be taken into consideration. However, the case of rPVB concentrations exceeding 8 wt.% results in forming microfibers, thus losing the advantages of nanofibrous webs. This is the reason why a concentration of 8 wt.% for rPVB was solely taken as a basis for all blended PVB solutions. While concentrations 8 and 10 wt.% of vPVB still exhibit singularities, their blends with 8 wt.% rPVB are already qualitatively acceptable (see Figure 6). The best results are obtained for the weight ratio 2:1 with a higher participation of rPVB.

**Figure 7:**
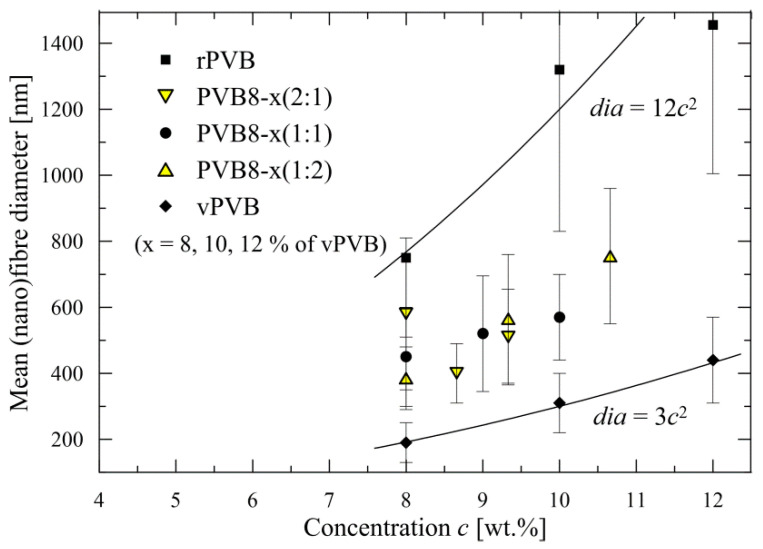
Mean nanofiber diameters for recycled, blended and virgin PVB.

**Figure 8:**
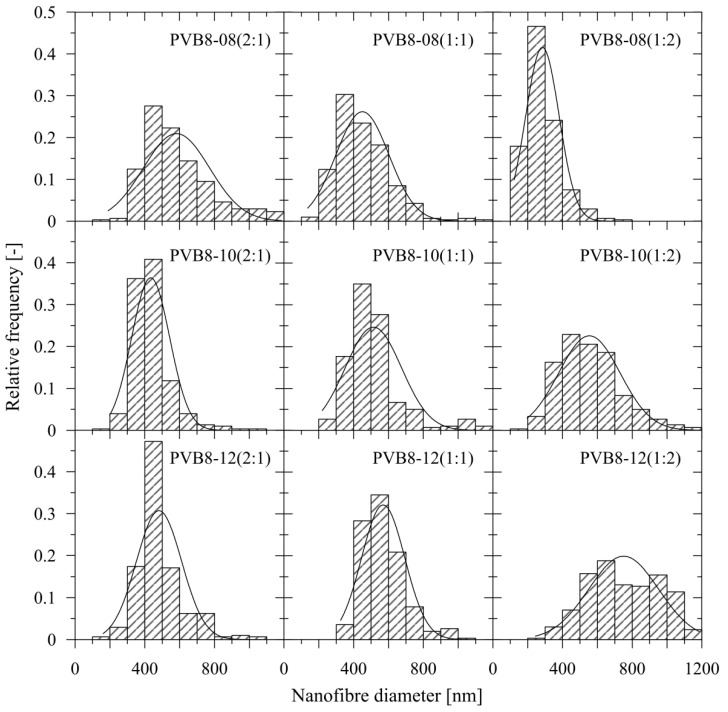
The histograms of the individual PVB blends with normal distribution.

Usage of only 8 wt.% solution of rPVB also guarantees a mean nanofiber diameter *dia* in the approximate range of 400–600 nm for all blended solutions, except of PVB8-12 (1:2), as depicted in Figure 7, and in more detail presented in Table 3, including standard deviations, with Figure 8 depicting the histograms. Each mean nanofiber diameter was derived from 300 measurements taken from three different images using the Adobe Creative Suite software (San Jose, CA, USA).

If the values for rPVB and vPVB nanofiber diameters are approximated by a power function (see Figure 7), a substantial difference among the recycled and virgin PVB as source materials for nanofibrous webs is apparent. A ratio surface/volume (≡π.*dia*/(π.*dia*^2^/4) per unit length) = 4/*dia* is approximately four times lower for recycled PVB in comparison with virgin PVB for all considered concentrations most probably due to presence of impurities in rPVB.

The presence of impurities (change in the chemical composition of employed polymeric materials) was described and proved by means of Fourier-transform infrared (FTIR) spectroscopy. FTIR spectroscopy was carried out to reveal the interactions among the recycled and virgin PVB, impurities (as e.g., plasticizers), and the solvent (ethanol) in the nanofibrous webs.

**Figure 9:**
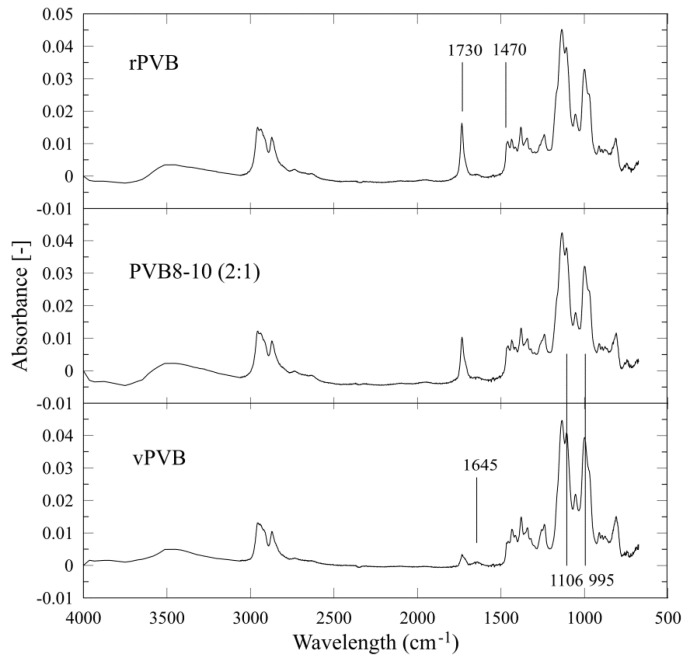
Attenuated total reflection FTIR spectra of the selected PVB nanofibrous webs.

The graphical results of measured spectra (between 4000 and 680 cm^−1^) of chosen samples (rPVB, PVB8-10 (2:1), vPVB) are depicted in Figure 9. The peaks at 1730 cm^−1^ are assigned to the C=O stretching vibration of the acetate group and the peaks at 1470 cm^−1^ and 1376 cm^−1^ are assigned to the C–H bending vibration [38,39]. The peaks at 1106 cm^−1^ and 1056 cm^−1^ are related to the butyral ring [40]. Other characteristic bands are summarized in Table 4.

The differences between the tested samples were found in the arising of peaks at 1730 and 1470 cm^−1^, and the decline of peaks at 1645, 1106, and 995 cm^−1^ with an increased amount of recycled materials standing for an increase of an ester and alkyl groups, with a corporate decrease of butyral rings in possible connection with plasticizers concentration growth. Noteworthy, this methodology proved that the relation between rPVB and vPVB weight ratios (2:1, 1:1, and 1:2) fairly corresponds to the change of the above-mentioned peak areas. In other words, the blends of rPVB and vPVB exhibited peak areas mutually shifted in dependence on the amount of contained rPVB, as presented for rPVB, PVB8-10(2:1), and vPVB, in Figure 7. These chemical changes also participate in increase of shear viscosity with an increasing portion of rPVB, see Figure 4.

**Figure 10:**
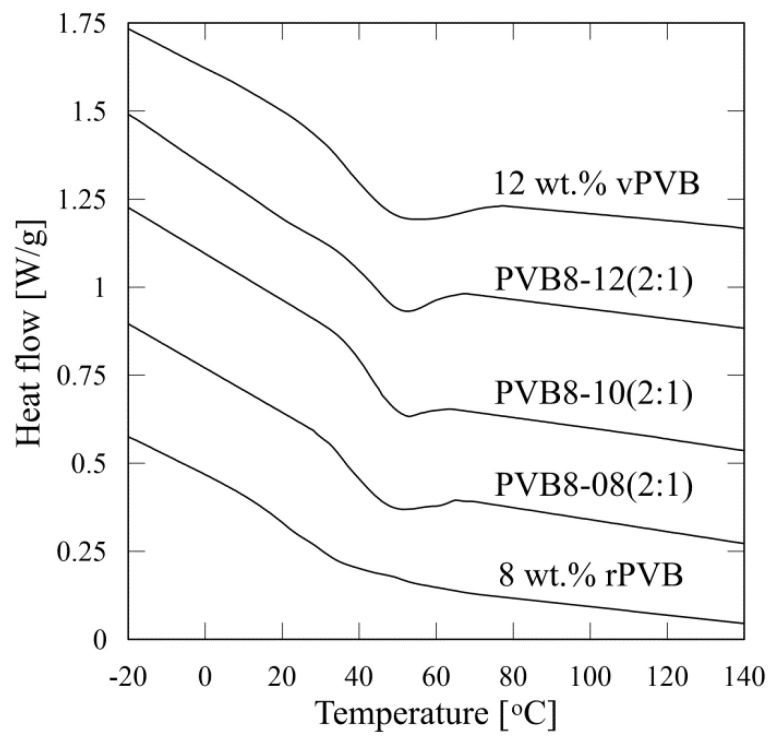
DSC thermograms of the tested PVB nanofibrous webs.

The brittleness of nanofibrous webs is bounded by glass transition temperature, *T*_g_, determined by differential scanning calorimetry. Applying a Mettler Toledo software STAR SW14 to a second heating scan, glass transition was evaluated from the midpoint of the heat flow change between the upper and lower tangents. The obtained DSC curves of the tested nanofibrous webs are depicted in Figure 10. The defined values of glass transition temperature are listed in Table 5.

Glass transition temperature *T*_g_ attains the values in the range of 27–47 °C depending on the PVB concentration and content of recycled material. A higher concentration of employed blended PVB solution shifts *T*_g_ to apparently higher temperature values (from 39.3 to 46.8 °C for concentration increased from 8 to 9.33%). An analogous increase is apparent with an increasing content of virgin material (26.7 °C for nanofibrous webs made of rPVB in comparison with 39.3 and 38.3 °C for webs made of PVB8-08(2:1) and vPVB, respectively).

The DSC thermograms, presented in Figure 10, prove a prominent lack of sensitivity for *T*_g_ evaluation of PVB nonwoven structures. Moreover, it is evident that the presence of recycled materials negatively affects the predicative ability of this experimental technique as a drop of heat flow is less distinctive. Since the temperature dependence of heat capacity of the tested nonwoven samples could not be enough to be accurately assigned with relevant significant changes in its thermal behavior around *T*_g_, temperature affected dynamic mechanical analysis was consequently carried out as a more sensible procedure.

**Figure 11:**
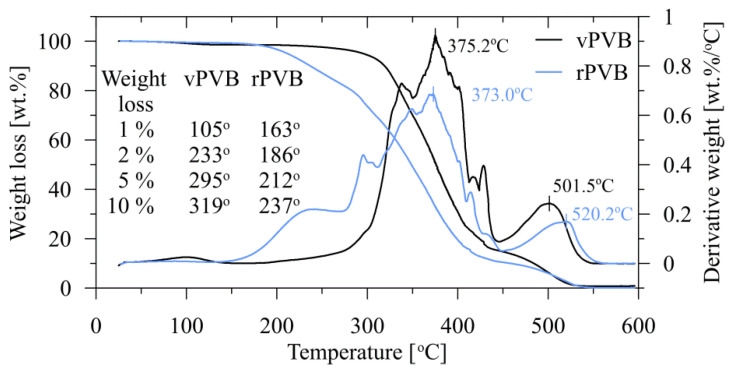
The results of thermogravimetric analysis and derivative thermogravimetric analysis for vPVB and rPVB.

The results of TGA and DTG analyses are shown in Figure 11. Relatively low material losses for lower temperatures are presented in the inserted table. For temperatures above 180 °C there is a significant difference in the mutual behavior of both materials. The steeper decrease in the weight loss of rPVB for temperatures exceeding 180 °C, in contrast to vPVB, can be attributed to a loss of plasticizer contents. A consequent course is an analogue to that described in [41,42]. There appears to be two weight-loss stages. The first one, between 280 and 420 °C, is characterized by the elimination of side groups, and the second one, between 450 and 530 °C, is where the fracture of the polymer (PVB) main chain takes place. As apparent from Figure 11, both samples (and analogously also the blended ones) exhibit very good thermal stability for temperatures up to the melting point.

**Figure 12:**
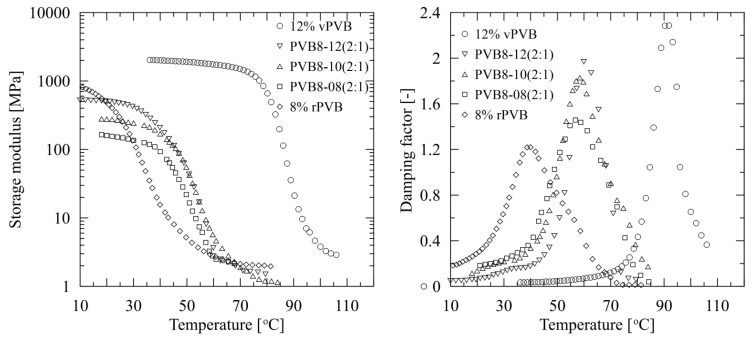
Temperature dependent behavior of storage modulus (**left**) and damping factor (tan delta) (**right**) of nonwoven rPVB and blended PVB nanofibrous webs, and vPVB film.

DMA analysis enables us to compare the viscoelastic properties of rPVB and blended PVB nonwoven webs, together with those of vPVB film, as presented in Figure 12.

In contrast to vPVB films, vPVB nonwoven webs testing is not presented as the nonwoven structure was much too brittle for dynamical mechanical experiments (already discussed above). In the graphs, the mechanical response of the tested materials to applied dynamic deformation amplitude is expressed through the temperature dependencies of the storage tensile modulus and loss factor (damping factor). Based on prominent changes in the mechanical behavior of the tested structures (both film and nonwoven webs), *T*_g_ was evaluated from the maximum of damping factor (tan delta, defined as a ratio of loss (*E*″) to storage (*E*′) tensile moduli). The determined values of *T*_g_ of the tested webs and film, together with loss factors, are summarized in Table 6.

Comparing the graphical results of the temperature dependent response of the damping factors of the tested materials obtained by the DMA technique (Figure 12), and numerically presented in Table 6, it can be deduced that the differences in glass transition temperature *T*_g_ are most probably influenced by a change of low molecular weight plasticizer content [43], rather than by a change of molecular weight. Namely, while the *T*_g_ of webs made of mere rPVB was determined as the lowest one, having a transition temperature close to 40 °C, the *T*_g_ of the tested vPVB film was defined as the highest one, slightly above 90 °C. This also reflects in the glass transition temperature for the blends of rPVB and vPVB. Its value systematically increases with an increasing participation of vPVB as simultaneously a content of plasticizers is decreasing. This is in compliance with previously presented results [44,45]. An increase in *T*_g_ is restricted by both limiting cases (for rPVB and vPVB), specifically, *T*_g_ shifts from approximately 56 °C to 60 °C. In this regard, dynamical mechanical analysis appears as a very effective method for plasticizer residues detection. In addition, the temperature effect on the mechanical response of plasticized materials is documented in Figure 12 (right), and also by shape variability of the loss factor peak, which is subject to plasticizer content. While for vPVB the peak is relatively thin and high, with the increasing contents of plasticizers a peak gets wider and lower. Regarding the tensile modulus of tested specimens, the highest level was reached by vPVB film followed by rPVB and blended PVB webs. Variations among blended PVB webs could be ascribed to the morphology of electrospun webs and their thickness determination.

**Figure 13:**
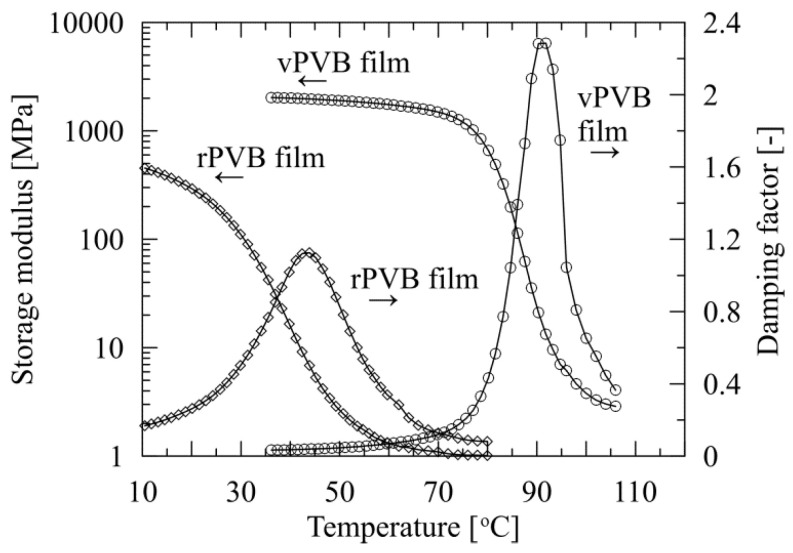
Comparison of temperature dependent behavior of storage modulus and damping factor (tan delta) of rPVB and vPVB films.

To describe variations of material characteristics of employed rPVB and vPVB, viscoelastic properties of films made of these two basic components were compared. The results in graphical form describing the temperature dependent behavior of storage and loss moduli of hot-pressed films made of virgin and recycled PVB, are presented in Figure 13, while the numerical results are introduced in Table 6.

The striking difference in the dependence of viscoelastic properties of rPVB and vPVB films on temperature is in compliance with the above presented results, as documented, for instance, for rPVB material; a glass transition temperature of 42.4 °C (evaluated from the maximum of damping factor) for rPVB film is very close to 39.9 °C defined for rPVB web.

**Figure 14:**
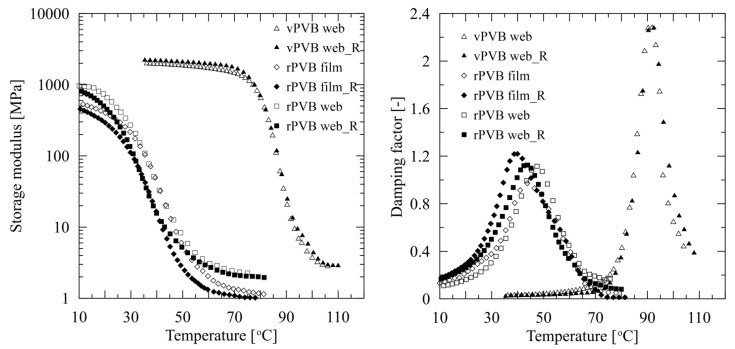
Comparison of temperature dependent behavior of storage modulus (**left**) and damping factor (tan delta) (**right**) of rPVB compressed films and electrospun webs.

Finally, a comparison of viscoelastic data evaluated for rPVB films and nonwovens webs, together with vPVB films from two subsequent tests of the same specimens, are presented in Figure 14. This documents the influence of the employed manufacturing and processing conditions on a change of mechanical performance of prepared products. The specimens used for the repeated experiments are denoted as _R. The second round of testing was performed with the aim to describe an influence of repeated heating process on the change of viscoelastic properties, potentially accompanied with material degradation, crosslinking, or plasticizer release.

The above presented experiments demonstrate that the temperature increase of vPVB (repeated heating of tested specimens to 110 °C) has a significant impact on the vPVB mechanical behavior. The determined increase of *T*_g_ by 0.5 °C thus confirms high reproducibility of the experimental method. On the other hand, the results of repeated experiments with rPVB specimens revealed that the *T*_g_ of both tested samples—webs as well as films—is slightly shifted to higher temperature (by approximately 3.5 °C) during the second heating scan. These results indicate that increased temperature participates in the slow release or decomposition of plasticizer rather than in molecular weight decrease. This causes a slight growth of complex modulus, together with an increase of glass transition temperature.

The transition temperatures of the nonwoven and film rPVB samples do not differ significantly. The glass temperature of the electrospun samples beside the pressed ones was found to be lower by about 4 °C, as documented in Table 7. This proves that the heating of rPVB during the production of manually pressed testing specimens also evokes plasticizer release, or its degradation.

These findings underline an important role of plasticizer residues in rPVB and blended PVB nonwovens. During the process of the electrospinning of rPVB and blended PVB solutions, the plasticizer is bonded to PVB and in this structure withstands inside the final nanofibrous webs. Hence, the plasticized PVB nonwovens can be tailored-made with respect to the pre-determined transition temperature (employing DMA technique).

**Figure 15:**
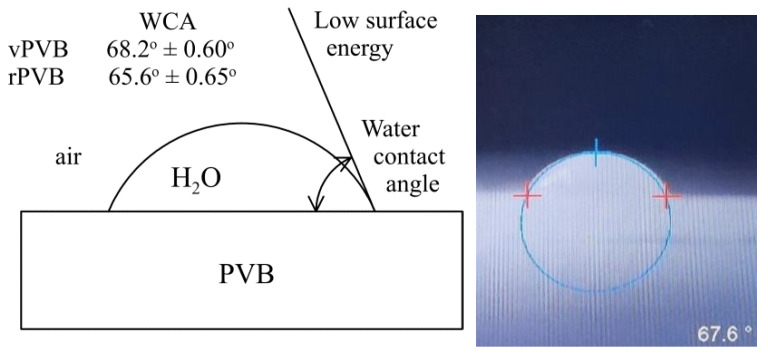
Water contact angle measurements (mean ± standard deviation) for vPVB and rPVB.

**Figure 16:**
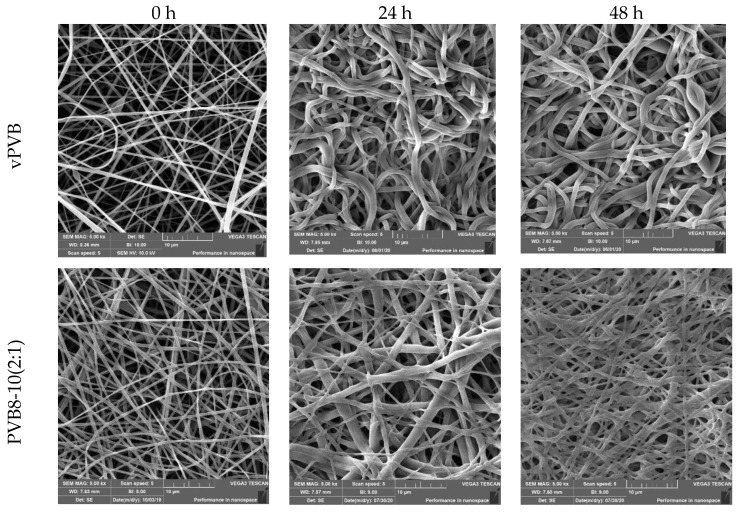

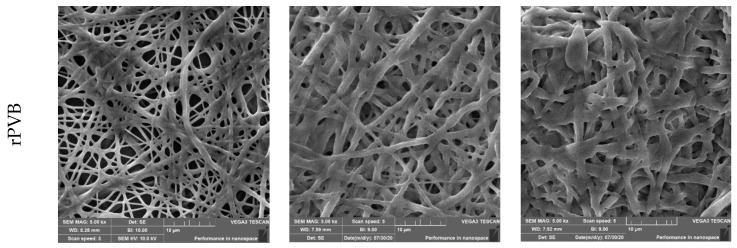
Morphology of electrospun mats after soaking in water (0, 24, and 48 h), scale length = 10 μm.

Plasticizers exhibit a minor role in the wettability of the individual samples where their participation influences the surface energy only moderately. For the evaluation of wettability, the water contact angle for both limiting cases was applied. Due to the low surface energy of vPVB, the mean contact angle attained 68.2°, which is in correspondence to the value 69.19° introduced in Yang et al. [46]. A very moderate increase in surface energy caused by a plasticizer presence was detected for recycled PVC, for which a value of the mean contact angle decreased to 65.6° only, see Figure 15. In both cases, 10 measurements were carried out. Dramatic changes in the morphology of the electrospun mats after soaking in water for 24 and 48 h are presented in Figure 16, documenting the presence of plasticizers.

Reducing the noise level is of great importance. The traditional study of sound absorbing materials is open to new possibilities with the onset of relatively new technologies—such as electrospinning. Used electrospun material, web morphology and layer thickness, and other properties, contribute to the sound absorption mechanism based on friction between the electrospun material and the air. This process is also strongly influenced by rheological properties of the used polymers, especially by viscoelasticity. Due to the viscous and thermal effects a portion of sound energy (classified as sound absorption coefficient) is converted into thermal energy. More detailed information on the usage of electrospun materials in sound absorbing can be found, e.g., in [47,48,49].

In the following we evaluate a usage of blended PVB nanofibrous webs as potential candidates for passive noise control. The efficiency of the individual materials strongly depends on the frequency of the emitted noise. For every frequency it is possible to set a so called sound absorption coefficient *α*, relating an amount of non-reflected sound energy to the incident sound energy [50]. Hence, the coefficient *α* attains a value within the interval [0, 1], where the values 0 and 1 correspond to two limiting cases: no absorption (*α* = 0) and full absorption (*α* = 1). To provide a better insight into the absorption efficiency of materials, the term noise reduction coefficient (NRC) was introduced by averaging four sound absorption coefficients at the octave band center frequencies 250, 500, 1000, and 2000 Hz and its rounding to the nearest multiple of 0.05. More detailed information is presented by sound absorption average (SAA) for the 12 1/3 octave frequency band ranging from 200 to 2500 Hz (200, 250, 315, 400, 500, 630, 800, 1000, 1250, 1600, 2000, and 2500 Hz), which is rounded to the nearest multiple of 0.01 [51]. These frequencies cover frequencies of typical human speech, but are quite limited compared to the human hearing frequency area (20–20,000 Hz). For instance, the third octave band around 4000 Hz is not used in SAA computation. Another 6-point criterion is based on averaging sound absorption coefficient at six frequencies: 125, 250, 500, 1000, 2000, and 4000 Hz ranging from relatively low frequency (125 Hz), but also covering relatively high frequency (4000 Hz) [52].

**Figure 17:**
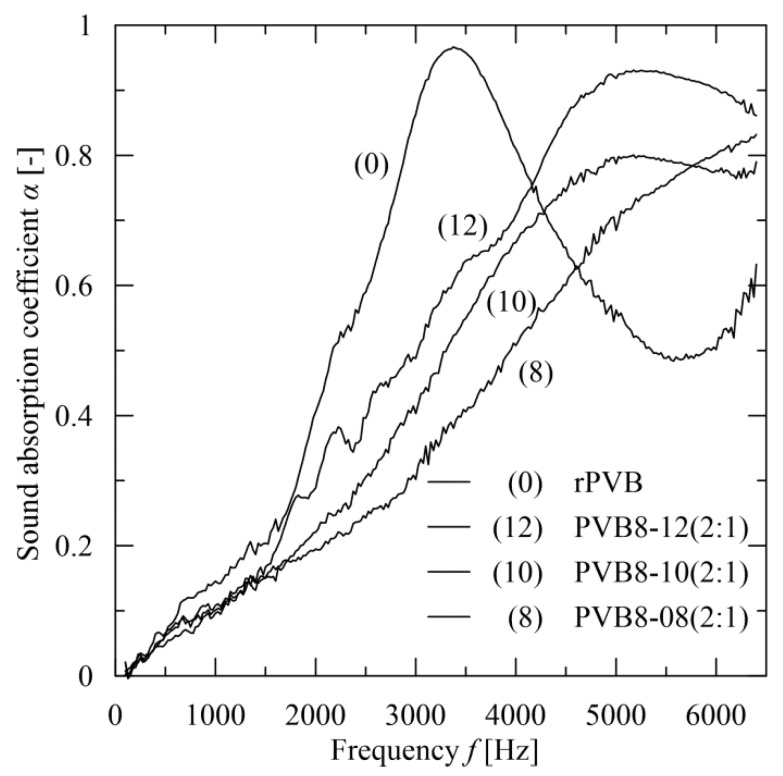
The courses of sound absorption coefficient *α* of chosen nanofibrous webs.

With respect to the preceding results and the applicability of recycled PVB, attention was focused to blended PVB nanofibrous webs with the prevailing participation of rPVB, specifically to PVB8-x(2:1), where x represents consecutively 8, 10, and 12 wt.% of vPVB. The sound absorption results are compared with the rPVB nanofibrous mat. A comparison with a vPVB mat is not presented due to the high fragility level of this mat for this type of evaluation, similarly to DMA testing observation. The basic morphological characteristics are summarized in Table 8 (for a determination of pore sizes the procedure presented in Sambaer et al. [53] was used) and the measured sound absorption coefficient *α* in the range 100–6400 Hz for the webs of approximate thickness 67 µm is presented in Figure 17. In contrast to a course of sound absorbance for rPVB (having absorption peak close to 1 around 3400 Hz), the blended PVB webs exhibit a slight decrease of the peak level, nevertheless with an evident tendency to be moved to higher frequencies, thus offering tunability of their sound absorption performance. It should be noted that the blended PVB webs sound absorbance dominates within the range above 4500 Hz, where a peak position is shifted to higher frequency with lower but wider maximum level for lower rPVB concentrations.

The value 0.2 is taken as a good limit for sound absorbing material [51]. The parameters NCR, SAA, 6-point, and maximal sound absorption coefficient α are listed in Table 9. It implies that PVB 8-12(2:1) mats attain this value for 6-point criterion. In a point-wise course, this value is exceeded by these webs for frequencies consecutively starting from the level of 1600 Hz. This provides a better result than is exhibited for instance by unpainted concrete block (α = 0.3 for frequency 4000 Hz), brick (<0.1 for all frequencies), gypsum wallboard (<0.1 for all frequencies) and at frequency of 4000 Hz fully comparable with acoustical plaster (*α* = 0.7) according to Hall ([54], Table 15.1). Comparing the results with the rPVB mats, the rPVB mats dominate with sound absorbance up to roughly 4000 Hz, where a dominant position takes over PVB 8-12(2:1) mats.

## 4. Conclusions

The permanent problem of a deposition of wasted windscreens of end-of-life vehicles represents a real ecological burden. This also concerns the PVB foils used in the windscreens. Their re-processing now becomes more attractive as the former financial imbalance between PVB recovery and newly fabricated PVB material is outweighed by new factors, such as the increase of oil prices, an increase of landfill fees, and environmental demands. In addition, among the processes using recycled PVB the electrospinning method is at the lower end of the cost spectrum, thus underlying production effectiveness.

The present contribution presents a possibility to use rPVB for the preparation of sound absorbing material. The basic characteristics as a noise reduction coefficient and sound absorption average do not exhibit remarkable sound absorption efficiency in the range of lower frequencies. However, the sound absorption of electrospun blended PVB webs guarantees not only a very promising level of noise reduction, but moreover offers a possibility for its frequency tuning being extended, and thus offers very good applicability for sound absorption in the frequency range from 3000 to 5000 Hz.

As follows from above, the application of these mats is focused on the fields with dominant middle and high frequencies (as parts of interior panels in transport means, parts in ventilation systems, as passive insulation middle frequency devices). Sound which is transmitted and impacts on the layer of rPVB material is absorbed with higher sound absorption coefficient α > 0.6 (in the technical application sound absorption coefficient α above 0.6 are classified as very good absorbers). These results are exhibited in a middle frequency area (from 4000–6500 Hz for the samples PVB8-08, PVB8-10 and PVB8-12). Specific results are provided by rPVB where one peak of sound absorption coefficient was found and is located in the frequency area between 2500–4000 Hz. However, the best results are provided by PVB 8-12(2:1).

Beside the proposal of the potential utilization of PVB scrap, it was documented that the electrospinnability of PVB solution is efficiently controlled through the monitoring of the employed solution viscosity independently of PVB molecular weight or impurities content. Moreover, the temperature dependent response of the mechanical properties of prepared electrospun webs, namely their glass temperature transition, can be customized altering rPVB and vPVB ratio; in other words, using plasticizer included in rPVB scrap.

The primary aim of this contribution—to recycle PVB from end-of-life vehicle windscreens, helping to reduce environmental burden- was confirmed. The consequent open questions are, how to optimize blended PVB nanofibrous layers from the point of view of their processing. The passage and absorption of sound energy through these materials is closely connected with a mean nanofiber diameter and surface density. While a diameter can be relatively easily modified by altering the entry process parameters (voltage, tip-to-collector distance, humidity, etc.) with the preserving nanofiber surface quality, changing the surface density is much more complicated [55]. Both of these parameters significantly contribute to determine the sound absorption coefficient through the specific surface area (enlarging with a decreasing mean diameter), the sizes of pores (increasing with an increasing mean diameter), and the overall porosity. To achieve an even distribution of the pores is also a subject of interest. As evident in [56], the sound absorption of a single electrospun layer can be enhanced by an application of multilayer nanofibrous membranes. Very good results can also be obtained by the use of composite materials when recycled PVB membranes are combined with classical sound-absorption materials, thus improving their efficiency. In this context, a thickness—affecting the distance of propagating sound waves within the nanofibrous mats—ranges to critical parameters. Its upper boundary can be limited by the space limits.

Tailoring the blended PVB nanolayers is substantially a factor of the range of wave frequencies faced with the absorbing material. This determines an optimization of morphology, chosen thickness, required mechanical and rheological properties. The above results show that blended PVB material is a good candidate for this optimization.

## Figures and Tables

**Table 1:** Various applications of rPVB.

Used Material	Application	Reference
PP/rPVB	a higher impact strength compared to the pure PP, improvement of the impact resistance of brittle polymers	Zanjanijam et al. [17,18]
PVC/rPVB	flooring applications	Bendaoud et al. [19]
modified rPVB potassium polytitanate	improvement of mechanical properties of composite coatings	Burmistrov et al. [20]
rPVB as a carbon source	prepare silicon carbide nanocrystals and carbon-coated Si as an anode for lithium ion batteries	Park et al. [21], Park et al. [8]
rPVB	an efficient toughening agent of Nylon 6	Cha et al. [22], Lee et al. [23]
rPVB	improvement of toughness of polyamide 6	Zanjanijam et al. [6], Valera and Demarquette [24]
rPVB/polyurethane	improvement of pure polyurethane properties	Sonego et al. [25]
rPVB/polyurethane	shape memory	Tsonev et al. [26]
rPVB/leather	analysis of sheet composites	Ambrosio et al. [27]
rPVB	used for yarn coating	Brendgen et al. [28]
rPVB/vinyl trimethoxysilane silanation	improvement of solvent resistance to organic solvents	Sonego et al. [12]

**Table 2:** Prepared bPVB made of rPVB and vPVB ethanol solutions.

Sample Code	rPVB Concentr. [wt.%]	vPVB Concentr. [wt.%]	Weight Ratio [-]	Final Solution Concentration [wt.%]
PVB8-08(2:1)	8	8	2:1	8.00
PVB8-08(1:1)	8	8	1:1	8.00
PVB8-08(1:2)	8	8	1:2	8.00
PVB8-10(2:1)	8	10	2:1	8.66
PVB8-10(1:1)	8	10	1:1	9.00
PVB8-10(1:2)	8	10	1:2	9.33
PVB8-12(2:1)	8	12	2:1	9.33
PVB8-12(1:1)	8	12	1:1	10.00
PVB8-12(1:2)	8	12	1:2	10.66

**Table 3:** Mean nanofiber diameters (including standard deviations) of recycled, blended and virgin PVB nanofibers (x = 8, 10, 12).

wt.%	rPVB [nm]	PVB8-x(2:1) [nm]	PVB8-x(1:1) [nm]	PVB8-x(1:2) [nm]	vPVB [nm]
8	750 ± 240	585 ± 200	450 ± 150	285 ± 95	190 ± 60
8.66		435 ± 110			
9			520 ± 175		
9.33		485 ± 135		565 ± 195	
10	1320 ± 490		570 ± 130		310 ± 90
10.66				755 ± 205	
12	1455 ± 450				440 ± 130

**Table 4:** Characteristic FT-IR spectra of PVB.

Wavenumber [cm^−1^]	Characteristic Absorption Bands of the PVB Based Polymer
1760–1640	C=O stretching of carboxyl-C and evidences of ketones and esters
1645	C=O conjugated and quin ketones
1470, 1376	C–H bending vibration
1106, 1056	C–O–C stretching vibrations of acetal group and hexatomic cyclic acetal group
1240, 995	C–O–C stretching vibration of acetate group
880–680	C–H out-of-plane bending

**Table 5:** Evaluation of *T*_g_ from DSC measurements of PVB webs.

Sample Code	Final Solution Concentration [wt.%]	*T*_g_ [°C]	Change of Heat Flow [J/g.K]
rPVB	8.00	26.7	0.44
PVB8-08(2:1)	8.00	39.3	0.56
PVB8-10(2:1)	8.66	45.8	0.62
PVB8-12(2:1)	9.33	46.8	0.30
vPVB	12.00	38.3	0.58

**Table 6:** Evaluation of *T_g_* from DMA measurements for rPVB and blended PVB nonwoven nanofibrous webs, and rPVB and vPVB films.

Sample Code	Tested Sample form	Final Solution Concentration [%]	*T*_g_ [°C]	tan(delta) [-]
rPVB	film		43.8	1.14
rPVB	web	8.00	39.9	1.22
PVB8-08(2:1)	web	8.00	56.8	1.46
PVB8-10(2:1)	web	8.66	58.4	1.82
PVB8-12(2:1)	web	9.33	59.9	1.97
vPVB	film		92.5	2.29

**Table 7:** Evaluation of *T*_g_ of rPVB and vPVB films using DMA measurements.

Sample Code	Tested Sample form	Final Solution Concentration [%]	*T*_g_ [°C]	tan(delta) [-]
rPVB	film		39.9	1.2
rPVB_R	film		43.4	0.9
rPVB	web	8	43.8	1.1
rPVB_R	web	8	47.4	1.1
vPVB	film		91.8	2.3
vPVB_R	film		92.4	2.3

**Table 8:** Basic morphological characteristics of chosen nanofibrous webs.

Sample Code	Mean Fibre *dia* [nm]	Mean Pore Size [µm]	Maximum Pore Size [µm]
rPVB	750 ± 240	2.08 ± 1.02	5.6
PVB8-08(2:1)	580 ± 230	1.95 ± 0.38	2.7
PVB8-10(2:1)	400 ± 90	0.94 ± 0.30	1.5
PVB8-12(2:1)	510 ± 145	1.35 ± 0.40	2.1

**Table 9:** The values of sound characteristics for chosen nanofibrous webs.

Sample Code	NRC [-]	SAA [-]	6-Point [-]	*α*_max_ [-]
rPVB	0.15	0.16	0.24	0.97 at 3376 Hz
PVB8-08(2:1)	0.10	0.10	0.15	0.83 at 6392 Hz
PVB8-10(2:1)	0.10	0.10	0.18	0.80 at 5176 Hz
PVB8-12(2:1)	0.10	0.13	0.20	0.93 at 5288 Hz

## Data Availability

All the experimental data are graphically presented or tabulated in this contribution.
